# Multiple disadvantages: class, social capital, and well-being of ethnic minority groups in the UK during the COVID-19 pandemic

**DOI:** 10.3389/fsoc.2024.1215676

**Published:** 2024-01-18

**Authors:** Yaojun Li, Lin Ding

**Affiliations:** Department of Sociology, School of Social Sciences, Manchester University, Manchester, United Kingdom

**Keywords:** ethnicity, class, social capital, well-being, COVID-19, UK

## Abstract

**Introduction:**

The COVID-19 pandemic has caused untold damage to the socio-economic lives of people all over the world. Research has also demonstrated great inequality in the pandemic experience. In the UK as in many other countries, people from ethnic minority backgrounds and in working-class positions have suffered disproportionately more than the majority group and those in salariat positions in terms of income loss, financial difficulty, and vulnerability to infection. Yet little is known about how people coped in the daily lives and tried to maintain their well-being during the most difficult days of the pandemic through social capital.

**Methods:**

In this paper, we draw data from the COVID-19 Survey in Five National Longitudinal Studies to address these questions. The survey covered the period from May 2020 to February 2021, the height of the pandemic in the UK. It contains numerous questions on contact, help and support among family, friends, community members, socio-political trust, and physical and mental health. We conceptualise three types of social capital and one type of overall well-being and we construct latent variables from categorical indicator variables. We analyse the ethnic and socio-economic determinants of the three types of social capital and their impacts on well-being.

**Results:**

Our analysis shows that social capital plays very important roles on well-being, and that ethnic minority groups, particularly those of Pakistani/Bangladeshi and Black heritages, faced multiple disadvantages: their poorer socio-economic positions prevented them from gaining similar levels of social capital to those of the white group. However, for people with the same levels of social capital, the effects on well-being are generally similar.

**Discussion:**

Socio-economic (class) inequality is the root cause for ethnic differences in social capital which in turn affects people’s well-being.

## Introduction

The coronavirus pandemic (COVID-19) that hit the world at the beginning of 2020 has caused unprecedented damage to people’s lives. It is estimated that the economic cost would go over $10 trillion (Ahmed et al., 2020). Millions of people lost their jobs and were plunged into poverty. An untold number of families lost their loved ones and billions of people were affected with the virus, many with severe long-term health consequences. The pandemic also exacerbated socio-economic inequalities, with those in prior disadvantaged employment and occupational positions and coming from ethnic minority heritages suffering more economic losses, virus infections and mortalities (Coates, 2020; Hu, 2020; Laurencin and McClinton, 2020; Topriceanu et al., 2020; Platt, 2021).

Research has shown that people of ethnic minority heritages in the UK have for decades suffered from racial discrimination in the labour market. They have more difficulty in finding a job (Heath and Di Stasio, 2019), are more likely to face unemployment during recessions (Li and Heath, 2010) and have slower career progression even when they have higher levels of educational qualifications (Li and Heath, 2018; Li, 2021). During the COVID-19 crisis, they were particularly hard-hit as they were more likely to lose their jobs, less likely to enjoy employment protection such as furlough (Hu, 2020; Brewer et al., 2021); and were more vulnerable to the virus infection (Pareek et al., 2020; Maddock et al., 2021). Some ethnic groups such as Indians are more likely to be key-workers: they make up 3% of the working-age population in England and Wales, but constitute 14% of doctors (Platt and Warwick, 2020). Similarly, relative to the white population, Black Caribbean women are more likely to work as NHS nurses and Black Caribbean men are more likely to work as bus drivers; Black African women are more likely to work as care workers, and Pakistani/Bangladeshi men are more likely to work as taxi-drivers (Bhala et al., 2020; Laurencin and McClinton, 2020; Platt, 2021). These characteristics make them more susceptible to multiple disadvantages induced by the COVID-19. Furthermore, ethnic minority members, particularly the first generation from non-English speaking countries, tend to have poor education, poor English, poor health and poor socio-economic position. All this and other related factors would affect their ability to conduct social activities with other community members, especially with those from the mainstream population, hence reduce their social capital. The deficiencies in socio-economic conditions and in social capital would further limit their ability to understand and access online service, such as using virtual devices to access income protection insurance and flexible workplace support arrangements.^1^

The UK government implemented three national lockdowns to protect the people when the coronavirus became rampant: on 23rd March, 2020; 5th November, 2020, and 6th January 2021. Much research has documented the ethnic inequalities in unemployment risks, occupational profiles, hospitalisations and mortalities (Marston et al., 2020; Platt and Warwick, 2020; Platt, 2021), yet little is known about ethnic inequalities in social capital and well-being during the most difficult days of COVID-19.^2^ This research is aimed to make a contribution in this regard. We find that people of ethnic minority heritages, particularly those of Pakistani/Bangladeshi and Black origins, faced multiple disadvantages during the COVID-19: they had poorer class positions, lower incomes, greater likelihood of experiencing worse financial situations in the wake of pandemic outbreak and were more likely to have COVID-19 infections; moreover, they had lower levels of social capital in terms of social contact, social support and social trust, and poorer physical and mental wellbeing.

This paper is structured as follows. In the next section, we discuss how we conceptualise and measure social capital and well-being; then we present our analysis of the patterns and interrelations of the three types of social capital that we have constructed with well-being; we discuss the ethnic and socio-economic underpinnings of social capital and well-being in their gross and net differences; we then use the three types of social capital together with ethnicity and socio-economic attributes as exploratory factors to assess their impacts on well-being. The paper will conclude with a discussion.

### Conceptualising and measuring social capital and well-being

There has been a huge debate on social capital in the last two decades with thousands of papers and dozens of books published, and there is no need to give a detailed account of the various strands of the theory here (see Li, 2015 for a review; see also Li et al., 2005, 2008 for discussions of conceptual and measurement issues of social capital). Briefly, there is a consensus among the leading scholars (Bourdieu, 1986; Coleman, 1988; Portes, 1998; Putnam, 2000; Lin, 2001) that social capital refers to resources residing in social networks. Scholars also agree that social capital plays a mostly positive role in a wide range of social life, from educational performance, children’s welfare, economic prosperity, crime prevention, democracy to health and happiness even though there is also a “dark side” of it (Portes, 1998; Putnam, 2000). The role of social capital is also acutely recognised in the COVID-19 combatting activities (Marston et al., 2020). There is much research in the USA to show that social capital is closely related to citizens’ willingness to comply with the government’s control measures, hence with the infection rates in the different states, and research also shows that political trust is strongly related to people’s compliance with the lockdown measures in China (Wu, 2021). Yet to the best of our knowledge, there is little research on how people create different domains of social capital to maintain their physical and mental well-being to get on and get ahead during the most difficult times of COVID-19 and how ethnic and socio-economic differences impact on the social capital and well-being profiles in the UK.

In this paper, we use social capital in a more limited but finer-tuned way than that in Putnam (2000), as practical help or affective/emotional support generated through informal social networking among family, friends, community members for the companionship, trust and socio-psychological well-being to get on and get ahead in the daily lives during the COVID-19 period. This is more like Putnam’s (2000) bonding or Lin’s (2001) affective social capital although bridging or linking functions of social capital (Woolcock, 1998; Putnam, 2000) are not excluded. We use the COVID-19 Survey in Five National Longitudinal Studies to construct measures of social capital and well-being. The respondents were recruited from participants of the five national representative cohort studies: the MRC National Survey of Health and Development (NSHD) for people born in 1946, the National Child Development Study (NCDS) for people born in one week in 1958, the 1970 British Cohort Study (BCS70) for people born in one week in 1970, the Next Steps (NS) for people born in England in 1989–1990, the Millennium Cohort Study (MCS) for people born in 2000–2002, and the parents of the MCS cohort members. Overall, there are 67,527 respondents in the survey. The ages of the survey respondents ranged from 19 to 74 (see Brown et al., 2020 for further details of the survey).^3^

The survey was conducted in three waves. The first wave was conducted soon after the COVID-19 pandemic outbreak, in May 2020, which was followed in wave 2 in September 2020, and then in wave 3 in February 2021. During this short period of time, three national lockdowns were enforced as previously noted along with various other measures of social distancing and restrictions stipulated by the Government (Institute for Government, 2022). This is therefore the period with the greatest challenge for most people in the UK. Given the severity of the pandemic situation, normal face-to-face engagement in formal civic activities was hardly feasible or permissible. Correspondingly, no data of formal involvement in civic organisations were collected in any of the three waves of the survey, and thus formal civic engagement will not form part of social capital conceptualisation and measurement as usually found in social capital literature (Putnam, 2000; Li et al., 2003, 2005). This notwithstanding, there were plenty of measures collected in the survey on informal sociability, ranging from helping, giving, contacting with kin and friends who live outside of the household, to various kinds of social support received and trust in generalised others, and in government and political parties, along with a range of physical and mental health indicators. There are thus ample measures on social capital and well-being which would allow us to conduct a good analysis of the ethnic and socio-economic differences in access to social capital and the role of social capital on well-being during the pandemic. Our main purpose in this paper is to try to see how the different domains of social capital are related to one another, how they are determined by socio-economic and demographic attributes, and how they in turn impact upon people’s well-being. With this in mind, we focus on the indicators of social capital and well-being that were asked in all three waves so as to maximise the sample size for the analysis. In other words, questions that were asked in a single wave but not across all three waves were not used in this analysis.

We focus on three domains of social capital (social contact, social support and social trust) and one domain of physical and mental well-being.

### Social contact

As social capital refers to resources that reside in social connections (Portes, 1998; Putnam, 2000; Lin, 2001; Li et al., 2005), social contact is the foundation stone of social capital. We use five items in the social contact section in the survey on the amount and kind of social contact that our respondents had with other people who did not live with them in the preceding week: (1) “In the last 7 days, on how many days did you meet up in person with any of your family or friends who do not live with you?”, (2) “In the last 7 days, on how many days did you talk to family or friends you do not live with via phone or video calls?”, (3) “In the last 7 days, on how many days did you keep in contact with family or friends you do not live with by email or text or other electronic messaging?”, (4) “In the last 7 days, on how many days did you take part in an online community activity, e.g., an online community group, online chat group, street or neighbourhood social media group?”, and (5) “In the last 7 days, on how many days did you give help to people outside of your household affected by Coronavirus or the current restrictions? Please include doing shopping, collecting medicines, checking in on people and any other voluntary work for community groups or other organisations.” The response modes are: (1) “Every day”, (2) “4–6 days”, (3) “2–3 days”, (4) “1 day”, and (5) “Never”. These are reverse-coded so that the higher numbers indicate greater contact and support provided, hence greater social capital generated.

### Social support

Social support refers to resources embedded in and mobilizable from one’s close social networks such as family, friends or community members, resources crucial in our daily lives and even more valuable during the COVID-19 times. In the survey, the respondents were asked the extent to which each of the following statements describes their current relationships with other people: (1) “I have family and friends who help me feel safe, secure and happy”; (2) “There is someone I trust whom I would turn to for advice if I were having problems”; and (3) “There is no one I feel close to”. The response modes are (1) “Very true”, (2) “Partly true”, and (3) “Not true at all”. The first two items are again reverse-coded for conceptual consistency with the contact series. In addition, the respondents were asked “If you were sick in bed how much could you count on the people around you to help out?” and “If you needed to talk about your problems and private feelings, how much would the people around you be willing to listen?” For these questions, the response modes are (1) “Not at all”, (2) “A little”, (3) “Somewhat”, and (4) “A great deal”. These items are also reverse-coded for consistency.

### Socio-political trust

In addition to the social contact and the social support items as discussed above, we also included two trust questions: trust in fellow citizens and trust in government and political parties. While there is debate in the literature as to whether social trust is an antecedent or component or consequence of social capital (Coleman, 1988; Putnam, 2000; Li et al., 2005; Uslaner, 2008), there is research to show that social trust captures an important part of social capital, as an expression of one’s outlook and reflection of self-confidence at the personal level or an indicator of social solidarity at the collective level which would play an important role in implementing government control measures in both Western countries and China (Wu, 2021). As for the generalised trust, the question reads: “On a scale from 0–10 where 0 means you are “not at all trusting” of other people and 10 means you are “extremely trusting” of other people, how trusting of other people would you say you are?” As for political trust, the question reads: “On a scale from 0–10 where 0 means you are “not at all trusting” and 10 means you are “extremely trusting”, how trusting are you that British Governments, of any party, place the needs of the nation above the needs of their own political party?” Together, the three sets of questions on contact, support and trust would give a strong representation of social capital, namely, “social networks and the norms of reciprocity and trustworthiness that arise from them” (Putnam, 2000: 19). We use them first as outcome variables underpinned by ethnic and other socio-economic groups, and then as explanatory variables on people’s well-being.

### Well-being

As for the well-being variable, we use a set of questions on people’s perceived physical health and mental well-being. As for the overall health status, the respondents were asked: “In general, would you say your health is – excellent, very good, good, fair, or poor?” As for mental health, there is a set of “loneliness” questions included: (1) “How often do you feel that you lack companionship”?, (2) “How often do you feel left out”?, (3) “How often do you feel isolated from others”?, and (4) “How often do you feel lonely”? The response modes are “Hardly ever”, “Some of the time”, and “Often”. Thirdly, we include a measure of life satisfaction: “Overall, how satisfied are you with your life nowadays?” In quality-of-life studies, outcomes like these are often used as well-being indicators (Diener et al., 1999; Easterlin, 2009; Li, 2016; Zhao et al., 2017; ONS, 2023).

These indicator variables provide a unique source for social capital and well-being research but due to space limit, we cannot use them as individual outcomes in a single paper. Given this, we extract the latent propensities for these domains along the conceptual lines discussed above. As shown in Table 1, our indicator variables are of categorical/ordinal/continuous natures with missing values. Also noted here is the fact, as shown in (2) of Table 1, that there were three social-support questions that were not asked of NCDS and BCS respondents. The values of these variables for the two cohorts were thus set as missing. Yet, the respondents of the two cohorts did answer the other questions in the series. This kind of situation where indicator variables are categorical and have varying amounts of missing data is quite common in social surveys which renders it inappropriate to use the conventional factor analysis requiring continuous indicator variables. Given this, we use two-parameter item response theory (IRT) models (Lord and Novick, 1968) to obtain estimates of individual levels of social capital and well-being. The IRT models are designed for categorical/ordinal indicator variables with missing values. The sets of items (component variables for each type of social capital and well-being) were selected as likely indicators of the potentially distinctive facets of these entities. With indicators for the different facets of social capital and well-being, the original multi-category numerical codes were reordered to form natural ordered scales.

**Table 1:** Latent scores for items of the types of social capital and well-being.

	Number of categories	Loading	Standard error
**Social contact with people outside household in last 7 days**			
Talking to family/friends via phone or video calls	5	1.000	(−)
Keeping in contact with family/friends by email etc.	5	0.976	0.015
Taking part in an online community activity	5	0.492	0.010
Giving help to people affected by COVID-19 restrictions	5	0.486	0.010
Meeting up in person with family/friends	5	0.281	0.008
Latent variable variance0.520 (0.009) *z* = 60.41			
**Social support from family, friends, and community members**			
Having family & friends who help me feel safe and happy^a^	3	1.000	(−)
Having someone I trust for advice if I have problems^a^	3	1.008	0.008
Having people I feel close to^a^	3	1.011	0.009
Could count on people to help out if I am sick in bed	4	0.860	0.007
Having someone willing to listen to my problems and private feelings	4	1.039	0.008
Latent variable variance0.668 (0.009) *z* = 75.21			
**Socio-political trust**			
Trust in other people^b^	10	1.000	(−)
Trust in British Governments or political parties^b^	10	0.675	0.011
Latent variable variance0.458 (0.009) *z* = 57.36			
**Well-being**			
Overall life satisfaction^b^	10	1.000	(−)
Overall health condition	5	0.510	0.006
How often do you feel that you lack companionship	3	1.242	0.005
How often do you feel left out	3	1.263	0.005
How often do you feel isolated from others	3	1.287	0.005
How often do you feel lonely	3	1.325	0.005
Latent variable variance0.488 (0.003) *z* = 145.82			

1. Items for “social contact” refer to variables *scon1-scon5*, those for “social support” to *socprov_a-c, sick, listen*, for socio-political trust to *trust* and *trustpolp*, and for “well-being” to *GHQ, satn, lonely_a–d*. Some of the variables were reverse-coded so that the higher values in the recoded variables indicate higher levels of social capital and well-being. Negative codes in the source variables (such as −8 for “Do not know” and −9 for “Do not want to answer”) were set as missing. For question wording, see text.2. The variables in the table were asked in all three waves but three items marked with ^a^ were not asked of NCDS and BCS respondents. Questions marked with ^b^ have 11 categories in the source variables but the first two categories (0 and 1) were combined as MPlus (8.2) allows an maximum of ten categories in operation. Source: the COVID-19 Survey in Five National Longitudinal Studies (the same for all tables in this paper).

The eventual indicators in each type were thus sets of ordinal/continuous variables. We then used two-parameter IRT models to obtain estimates of the underlying domains of social capital and well-being. An IRT postulates that a single continuous factor underlies responses to all items within a set but that this factor is “measured” subject to error by each item. A continuous score underlies each item, the sum of a true score contribution and an error. The distribution of this score is divided up by a set of ordered thresholds, with each distribution being associated with observing one of the possible responses. A respondent’s categorical score is therefore determined by their continuous score falling within a particular range of values defined by an adjacent pair of thresholds. Since the continuous score is not directly observable, it is commonly considered to be a latent variable.

However, different items within a set may have different item characteristics. Items are allowed to differ in two ways. Firstly, items may have different threshold parameters. This allows, for example, fewer people to say that they “meet up in person” than to say that they talk with family and friends via phone or video calls, which is highly understandable given the severity of the pandemic infection and the government measures of lockdown, social distancing and other preventive restrictions. Secondly, items may have different sensitivity or factor loading parameters. This allows items to be strongly or weakly related to the underlying factor, or correspondingly to vary in the extent to which they measure the underlying factor rather than something else. Choosing a proportional odds ordinal logistic parameterisation allows the model to be specified by


lnprYij<=kprYij>k=αiK+λiηj

where $Y_{ij}$ is the response to item *i* from individual *j*, $\eta_{j}$ is the score of individual *j* on the latent factor, $\lambda_{i}$ is the factor loading for item *i*, $\alpha_{iK}$ is the threshold for a response of *K* or above. For an item with *K* categories, 1 to *K*, $\alpha_{iK}$ = ∞. Standard identification restrictions are necessary and we estimate the variance of the latent variable but constrain the first factor loading to 1. All variables have to be measured in some units and this restriction merely implies that the latent variable is to be measured in the units of item 1. It is also usual to make some parametric assumption about the distribution of the latent variable in the population. We have assumed this to be normally distributed.

The models were estimated using MPlus (8.2). Respondents with partially incomplete sets of responses were included under the assumption of the missing data being missing at random (Rubin, 1976). Estimates of scores on the underlying factor for each individual were calculated using empirical Bayes’ methods. This provided estimates both of individual scores and of estimation precision, the latter tending to be lower for those with incomplete data. The analyses indicated that the items within each set were indeed associated with a single dominant underlying latent variable and that the latent variables were rather weakly correlated with each other. These conditions justified our approach of fitting IRT models to each item set separately.

The scores obtained for the three types of social capital were standardised with a mean of zero and standard deviation of one. This procedure ensures that we can directly compare the differences from one type to another when we assess the ethnic and other social determinants of social capital generation. In our analysis of the impacts of social capital on well-being, we put the standardised scores into quartiles so that we can see the effects more clearly.

### Explanatory variables

We use a range of socio-demographic including COVID-19 specific factors as determinants of social capital and well-being. Our key interest in this paper is to explore the ethnic differences in generating informal resources among family and friends so as to get on and get ahead during the pandemic period, and therefore gaining ethnic information is of crucial importance. As the COVID-19 Survey in Five National Longitudinal Cohort Studies did not ask questions on the respondents’ ethnic identity in any of the three waves, we traced the ethnic information from the linked cohort surveys. Because of the small sample sizes for certain ethnic groups such as Chinese, we coded five main ethnic groups: White, Black, Indian, Pakistani/Bangladeshi and Other.^4^ While it would be desirable to have a more detailed coding, there were limitations in the earlier cohorts, and our coding is more detailed than found in other studies using the COVID-19 data sources (Ahmed et al., 2020; Hu, 2020; Topriceanu et al., 2020; Maddock et al., 2021).

As the COVID-19 pandemic is a very challenging experience, pooling up family resources is of great importance. As people’s resources, particularly economic resources, are best captured in terms of occupational (class) positions (Goldthorpe and McKnight, 2006), we coded a class schema using the National Statistics Socio-Economic Classification (NSSEC): higher level professional-managerial (salariat) class, lower salariat, intermediate class of clerical workers, self-employed and manual supervisors, semi-routine, routine and workless. We used the respondent’s or, where missing, partner’s class, as family class position. As many respondents and their partners did not provide information on class (31%), we tracked their class information from the linked surveys. Most of the respondents who failed to provide class information are the oldest (NCDS) and the youngest (MCS) in the five cohorts, 47 and 66%, respectively. Given the relatively large size of missing cases, we assign a separate category called “Missing” in the tables. In addition, we also include information on changes of family financial situation before and after the pandemic outbreak, whether the respondent or partner was a key-worker (Department of Education, 2022), whether the respondent had COVID-19 and whether they had received a shielding letter warning them of severe risks of catching the virus due to their existing health conditions.

In the following, we report the results of the analysis. We start with the IRT modelling of social capital domains and well-being, discuss their correlations, then move to bivariate and multivariate analysis of the three types of social capital, and finally come to the assessment of well-being by social capital and ethnic and other socio-demographic attributes.

## Results

### Patterns of social capital and well-being

Table 1 gives parameter estimates for the three types of social capital and of well-being. Factor loadings greater than 1 indicate that the corresponding item is more strongly associated with the latent variable than is the reference item, namely, item 1. A factor loading close to zero (say less than 0.3) indicates that the corresponding item does not measure this particular latent variable well. In our case, only one indicator variable (meeting up in person with family or friends who do not live in the household) has a low but still highly significant factor loading (0.281). In the data, only 6.4% of the respondents responded that they met up in person their family or friends who did not live with them every day in the previous 7 days. This is as expected, as people were not supposed to meet up in person with others unless absolutely necessary under the special circumstances of lockdown or social distancing policies. On the whole, talking to and keeping in touch with family/friends via phone, video calls or emails were the most common methods of social contact used by the respondents. They were very strongly associated with the first latent factor, followed by online community activity and giving help to people affected by COVID-19 restrictions. Latent factors for social support and trust had very high loadings among the constituent indicators. Indeed, in all cases (with the possible exception of “meeting up in person”) there was strong evidence of association among the items within a set explainable by an underlying latent dimension of individual variation, and the *z*-statistics showed little variation between sets of items (*z* = 60.4, 75.2, and 57.4 for social contact, social support, and socio-political trust respectively).

For ease of presentation, we put the indicators for latent factor “well-being” in Table 1 as well. We can see that all indicator variables within this set load strongly. Interestingly, mental health (loneliness) indicators load much more strongly than do the general health question (GHQ) indictors. It is also the case that the *z* score (at 145.8) is much higher here than for the three social capital dimensions.

Table 2 shows the pair-wise correlations among the latent factors for social contact, support, trust, and well-being.^5^ We can see that social contact is weakly associated with the other three factors but social support is strongly associated with trust and well-being (0.63 and 0.72), and social trust is also strongly associated with well-being (0.62). It is also noteworthy that whilst the associations between social contact with the other three latent factors are rather week, they are nevertheless all positively associated. The associations suggest that people who make more contact with family, friends and community members are also more likely to get more social support that makes them feel safe and secure, to have people they can trust when they need advice for problems, with whom they feel close, who can offer much needed help when they are sick in bed and who are willing to listen to their problems and private feelings tend to have a more trusting outlook. This comports with observations that “thick”, bonding relations have special values in times of need (Putnam, 2000).

**Table 2:** Pair-wise correlations of types of social capital and well-being.

	1	2	3	4
1 Social contact	1.000			
2 Social support	0.420	1.000		
3 Socio-political trust	0.301	0.638	1.000	
4 Well-being	0.141	0.729	0.628	1.000

*N* = 67,421. All correlations are significant at the 0.001 level. Weighted analysis.

### Ethnic differences in socio-economic and COVID-19 conditions

Before we discuss the ethnic differences in social capital and well-being, it is necessary to have a brief look at their socio-economic and COVID-related situations. We present the information in Table 3. Here our data are consistent with much of the existing findings (Li and Heath, 2008; Hu, 2020; Platt, 2021). Thus, with regard to class, we find that the Black group is fairly close to the white group in access to the salariat (32 and 34% respectively), Indians are most likely and Pakistanis/Bangladeshis least likely to be in the most advantaged class (42 and 28% respectively).

**Table 3:** Ethnic differences in socio-economic and COVID conditions (percentage by column for each variable).

	Ethnicity					All
	White	Black	Indian	Pakistani-Bangladeshi	Other	
**Class**						
Higher salariat	13	10	19	10	12	13
Lower salariat	21	22	23	18	19	21
Intermediate	20	18	21	19	21	20
Skilled manual	10	9	6	9	10	10
Unskilled manual	5	6	2	4	5	5
Missing	31	35	29	39	33	31
(All)	100	100	100	100	100	100
**Financial change**						
Worse	29	32	27	39	36	29
Same	50	42	55	49	44	50
Better	21	25	18	12	19	21
(All)	100	100	100	100	100	100
**COVID-19 infection**						
No	88	85	84	78	87	88
Yes	12	15	16	22	13	12
(All)	100	100	100	100	100	100
**Keyworker status**						
Other	63	70	69	72	74	63
Yes	37	30	31	28	26	37
(All)	100	100	100	100	100	100
**Receiving shielding letter**						
Other	95	91	95	93	95	95
Yes	5	9	5	7	5	5
(All)	100	100	100	100	100	100

Weighted analysis and rounded figures.

Class position is closely related to the security of employment, stability and prospect of income; and it is also related to the family financial change before and after the onset of the COVID-19 pandemic. The Pakistanis/Bangladeshis are the poorest groups in the country, with 55.7% of the former and 48.7% of the latter as against 16.8% of the whole population being in poverty (Li, 2018), and they are also least likely to be in salariat positions offering stable incomes. It is painful to see that their financial situation has changed worse since the pandemic began, at 39%, relative to 29% for the whole sample or 27% for the Indian group.

Ethnic minority groups are also more likely to be affected by the COVID-19 virus. The UK’s Intensive Care National Audit and Research Centre reported on May 1, 2020, that people from ethnic minority groups were over twice as likely to suffer from COVID-19 infections as their general representation in the population (34% versus 14%; cited from Bhala et al., 2020). We also have this kind of information in our data. Respondents were asked whether they had got COVID-19 coronavirus. The question has four categories: 1 “Yes, confirmed by a positive test,” 2 “Yes, based on strong personal suspicion,” 3 “Unsure”, and 4 “No.” We follow the COVID-19 Survey in Five National Longitudinal Studies User Guide (p54) and combine the first two categories as “Yes” and the last two categories as “No.” The analysis shows that all main ethnic minority groups were more likely to report infection than white respondents: at 15, 16, and 22% for the Black, Indian and Pakistani/Bangladeshi groups, respectively, as compared with 12% for the white group. The Pakistanis/Bangladeshis were thus nearly twice as likely to be infected as the general population. The poor working environment (the men in these two groups were most likely to work as taxi drivers), multiple-generational living arrangement, and over-crowded living spaces were the most likely causes for the high rate of infection. Interestingly, people of ethnic minority heritages were less likely to work as key-workers and only people of Black and Pakistani/Bangladeshi origins were more likely than the white group to have received shielding letters, at 9, 7, and 5%, respectively.

### Ethnic and socio-economic impacts on social capital and well-being

Having looked at the overall contours of social capital and well-being and the ethnic differences in socio-economic and COVID-related situations, we now come to the main purpose of the paper and explore the ethnic and socio-demographic underpinnings of social capital and well-being. We first look at the determinants of social capital and then move to well-being where we also use the three types of social capital as explanatory variables. We shall first, in Table 4, look at the bivariate relationship between each explanatory variable and our social capital and well-being variables. To aid exposition, we present significance tests of the difference of means between each other category and the reference group in each explanatory variable, with the reference category shown in italics. Then we shall, in Table 5, conduct multivariate analysis where the effects of key independent variables will be simultaneously assessed. Finally, we shall, in Table 6, assess both social capital and socio-demographic effects on well-being.

**Table 4:** Standardised mean scores of types of social capital and well-being by socio-economic and COVID-19 factors.

	Social contact	Social support	Socio-political trust	Well-being	Approx. No.
**Ethnicity**					
*White*	−0.014	−0.028	−0.011	−0.006	58,490
Black	0.063	−0.161*	−0.374***	−0.224***	1,260
Indian	−0.013	−0.069	−0.093	−0.096	1,350
P/B	−0.103*	−0.323***	−0.203***	−0.305***	1,563
Other	−0.029	−0.196*	−0.163	−0.209*	1,193
**Gender**					
*Male*	−0.224	−0.081	−0.063	0.053	26,077
Female	0.156***	−0.030***	−0.021**	−0.113***	39,286
**Class**					
*Higher salariat*	−0.036	0.101***	0.106***	0.205***	9,589
Lower salariat	0.009**	0.058***	0.043***	0.100***	14,536
Intermediate	−0.013	0.002***	0.020***	0.062***	12,463
Skilled manual	−0.011	−0.151	−0.129	−0.183**	5,024
Unskilled manual	−0.075	−0.137	−0.098	−0.073	2,332
Missing	−0.049	−0.195	−0.182*	−0.246***	23,477
**Financial change**					
*Worse*	−0.024	−0.228	−0.197	−0.251	18,239
Same	−0.040	−0.024***	0.004***	0.038***	31,877
Better	−0.002	0.098***	0.048***	0.096***	15,326
**COVID-19 infection**					
*No*	−0.036	−0.055	−0.034	−0.022	58,861
Yes	0.056***	−0.044	−0.103**	−0.111***	8,033
**Keyworker status**					
Other	−0.018**	−0.101***	−0.087***	−0.117***	41,551
*Yes*	−0.040	0.025	0.025	0.105	25,870
**Receiving shielding letter**					
Other	−0.026	−0.043***	−0.037***	−0.016***	64,668
*Yes*	−0.027	−0.272	−0.197	−0.373	2,753

Class refers to respondent’s or, if missing, partner’s job. Where no class information is available, a workless category is assigned. Keyworker status refers to respondent or partner being a keyworker. Each of the other categories in a variable is contrasted with an italicised category, with the results of significance tests shown: ^*^p < 0.05, ^**^p < 0.01, and ^***^p < 0.001. Owing to the large amount of data presented, standard errors and the 95% confidence intervals are not shown but are available on request. Weighted analysis, the same below.

**Table 5:** Regression coefficients of social capital types by social and COVID-19 factors.

	Social contact	Social support	Socio-political trust
**Ethnicity (white = ref)**			
Black	0.040	−0.153*	−0.389***
Indian	0.015	−0.066	−0.091
P/B	−0.102*	−0.272***	−0.163**
Other	−0.024	−0.155	−0.138
**Sex (male = ref)**			
Female	0.400***	0.076***	0.064***
**Class (unskilled = ref)**			
Higher salariat	0.036	0.177***	0.130**
Lower salariat	0.041	0.134**	0.079
Intermediate	0.035	0.116**	0.071
Skilled manual	0.009	−0.036	−0.070
Missing	−0.031	−0.100*	−0.145**
**Financial change (worse = ref)**			
Same	−0.020	0.190***	0.194***
Better	0.028	0.277***	0.216***
Having got COVID infection	0.095***	0.012	−0.064**
Being a keyworker	−0.054***	0.036*	0.032*
Having received shielding letter	0.012	−0.174***	−0.106*
Constant	−0.224***	−0.272***	−0.178***
*R* ^2^	0.044	0.028	0.025
*N*	59,656	59,656	59,656

**Table 6:** Regression coefficients on well-being by types of social capital and socio-demographic factors.

	Model 1	Model 2	Model 3	Model 4
**Social contact (Bottom = ref)**				
Top	0.309***			−0.339***
2nd	0.313***			−0.270***
3rd	0.018			−0.149***
**Social support (Bottom = ref)**				
Top		1.983***		1.654***
2nd		0.968***		0.747***
3rd		0.736***		0.609***
**Social trust (Bottom = ref)**				
Top			1.563***	0.688***
2nd			1.089***	0.533***
3rd			0.711***	0.395***
**Ethnicity (White = ref)**				
Black	−0.232***	−0.087*	−0.034	−0.011
Indian	−0.094	−0.043	−0.034	−0.082***
P/B	−0.276***	−0.106***	−0.186***	−0.069***
Other	−0.201*	−0.131*	−0.124*	−0.117***
**Sex (male = ref)**				
Female				−0.155***
**Class (unskilled = ref)**				
Higher salariat				0.081***
Lower salariat				0.041*
Intermediate				0.059***
Skilled manual				−0.042*
Workless				−0.045**
**Financial change (worse = ref)**				
Same				0.111***
Better				0.074***
Having got COVID infection				−0.066***
Being a keyworker				0.064***
Having received shielding letter				−0.201***
Constant	−0.164***	−0.917***	−0.838***	−0.964***
*R* ^2^	0.026	0.486	0.322	0.567
*N*	63,856	63,856	63,856	59,656

We first come to the bivariate analysis between our explanatory and outcome variables. As the social capital and well-being variables are standardised with a mean of zero and a standard deviation of unity, the scores of different outcome variables for each explanatory variable can be directly compared. Take ethnic differences in social contact for example. We find that, with regard to social contact, only people of Pakistani/Bangladeshi heritages are less likely than white respondents to have contact with family and friends in the previous 7 days, and all other ethnic groups were fairly similar to the white group in this respect. But with regard to social support, social trust and well-being, all respondents in the ethnic minority groups were less likely, with those of Black and Pakistani/Bangladeshi origins being highly significantly so, than white respondents to have social support, to exhibit a trusting attitude towards fellow citizens, the government and political parties,^6^ and to have well-being. On the whole the data show that ethnic minority groups have lower levels of social capital and well-being.

We can also look at the gender and socio-economic differences in social capital and well-being at the bivariate level. As for gender, we find that women were significantly more likely than men to have contact with their family and friends but were less likely to have social support, to show social trust and to report similar levels of well-being. This echoes previous findings on trust by Li et al., (2005: 115), and it is a sad result that women provided so much more social contact and help but had much lower levels of well-being than did men.^7^

With regard to class and financial effects (as indicated change in financial status before and after the COVID-19 outbreak), we find a fairly clear gradient between economic advantage and social capital and well-being position. Apart from social contact where class differences are rather weak, we see strong class effects on social support, socio-political trust and well-being, with those in higher class positions having higher scores. For example, the differences between those in the higher professional-managerial salariat positions and their counterparts in the routine manual positions are 0.238, 0.204, and 0.278 in social support, social trust and well-being, which are all highly significant at the 0.001 levels. Similarly, as compared with those whose financial situation has become worse, those who feel that their situation has become better have scores 0.326, 0.245, and 0.347 higher, again all highly significant.^8^

As noted above, we have three COVID-19 specific variables: whether the respondents had been tested positive for COVID-19 infection, whether they were key workers and whether they had received shielding letters. As compared with those who had not experienced any infection, the respondents who were confirmed of infection or who had strong suspicion based on personal evidence that they had caught Coronavirus had higher levels of social contact, but scored negatively on social support, trust and well-being. Interestingly, key-workers scored higher than non-keyworkers on support, trust and well-being. Further reflection would suggest that this is not surprising. During the lockdown, there were around 7 million keyworkers in the country who went out to work in spite of the heightened risks. Keyworkers exhibited higher levels of social support and trust possibly because they realised that even though their work involved a greater risk, they were doing a highly valued and indispensable service keeping the country running whether they were doctors, nurses, teachers, food providers, social workers, carers or transport workers.^9^ The effects of shielding letter on social capital were generally weak although those on well-being were marked.

Having looked at the gross differences in the three types of social capital and well-being by ethnic and other socio-economic attributes, we now look at their net effects. The data are shown in Table 5. Here we find that when all other variables in the models were simultaneously controlled for, the net effects for the relevant variables and categories within the variables stay at a similar level to that found in Table 4 when only the bivariate analysis was conducted. Take ethnicity for example. The differences between people of Pakistani/Bangladeshi heritages and white respondents in the three domains of social contact are significant in Table 5 as they are in Table 4, which is also the case for the differences between Black and white respondents in social support and social trust. Thus with the exception of people of Indian heritage who show some weaker but non-significant levels of social capital than do white respondents, we find Black and Pakistani/Bangladeshi groups markedly and significantly less likely than their white peers to gain social support or to exhibit socio-political trust when all other factors in the models were taken into account. In the other respects, women, those in higher class positions and in better economic situations still reported higher levels of social capital, whether in terms of social contact, social support or socio-political trust. It is interesting to note that, other things being equal, women reported higher levels of social capital in all three domains net of all other factors controlled for in the models, a changed situation as compared with the gross analysis reported in Table 4.

With regard to COVID-19 specific variables, we find that, other things being equal, those who were infected with COVID-19 were more likely to have had contact with family, friends or community members than those who had had no COVID-19 infection, but less likely to hold trusting views of other people, government or political parties. In other words, other things being equal, having had coronavirus infection experience would have a negative impact on people’s outlook, a situation echoed by receipt of shielding letters but keyworker status would enhance trusting attitudes.

### Ethnicity, social capital, and well-being

Finally, in this section, we come to the core question in this paper: the role of social capital and ethnicity on well-being. We also wish to explore which of the three types of social capital we have delineated is of greater importance in promoting physical and psychological well-being that is crucial for people under the most challenging circumstances of our times, and the roles played by ethnicity and socio-economic factors.

Table 6 shows the effects of social capital, ethnicity, socio-economic and COVID-19 specific factors on well-being. In order to see the social capital effects more clearly, we put the standardised scores into quartiles. We report results from multivariate OLS regressions. Four models are constructed, with the first three models including each of the three social capital types, respectively, plus ethnicity, and model 4 pooling together all three domains of social capital, ethnicity and other socio-economic variables.

Table 6 shows that, when only each type of social-capital and ethnicity were included (Models 1–3), people with higher levels of social contact, social support and socio-political trust all showed significantly higher levels of well-being than those in the bottom quartile. Even though the coefficients for the higher levels of social contact are not large, they are nevertheless positive and highly significant. Looking at the coefficients for the other two types, it appears that social support has most benefit for people’s well-being, followed by socio-political trust. Take the top quartile in each domain for example. The coefficients are 0.309, 1.983, and 1.563 higher than for the bottom quartile in each domain, respectively. All higher levels in each variable have significantly higher scores than the bottom quartile (at the level of 0.001). The effects of social support and socio-political trust are noticeably more salient than those of social contact. Further analysis showed that even the third level of social support and socio-political trust has significantly higher scores than the highest level in social contact (at the level of 0.001) and that the coefficient of the top quartile in social support (1.983) is also significantly higher than the coefficient for the top quartile in socio-political trust (1.563), again at the level of 0.001. All this shows that social support at the time of COVID-19 is of crucial importance in maintaining and safeguarding people’s well-being. While social contact and trust also play indisputable roles, they cannot compare with social support.

We can also see that when the effects of the different domains of social capital are taken into consideration, there are significant ethnic differences except for the Black group under model 3. Compared with the white population, people from ethnic minority backgrounds were found doing significantly less well in their physical and mental health. It is of interest to compare the coefficient for the Black group in this table with that in Tables 4, 5 for the gross and net effects with regard to their trust levels. In Table 4, we found that they had a mean standardised score of −0.363, which is significantly lower than that by the white group and is the lowest of all five ethnic groups.^10^ Put differently, if the national level of socio-political trust were set at 100%, then the trust level by the Black group would be at around 64%. In Table 5, we found that people of Black origins are 0.389 points behind white people in trust in the net effect, that is, when the confounding factors are taken into account. Combining these factors, we may say that ethnic minority groups, black people in particular, tend to hold a less trusting attitude than white respondents, but for people with similar levels of trust, black and white groups are not significantly different in their well-being levels.

In Model 4 of Table 6, we put the three types of social capital, ethnicity and all other explanatory variables together in assessing their relative effects on well-being. The first impression that strikes us is that relative to findings in Models 1–3, the data under model 4 had their magnitudes weakened due to the inclusion of the other variables. Most strikingly, the coefficients for the three higher levels of social contact now become negative, and the coefficients for the three higher levels of socio-political trust were reduced to a half, and the ethnic effects were also reduced to varying degrees although the coefficients became significant. This is as expected as we are comparing like with like with many attributes taken into simultaneous consideration. It is revealing to see that social support and socio-political trust are still positively associated with well-being. The negative effects of social contact should be understood as suggesting that for those at similar levels of social support and social trust, and given all other factors in the model, the higher levels of social contact would have detrimental impacts on their well-being. Perhaps too much help and support to family, friends and community members during the COVID times made the givers exhausted and depressed.

Focusing on the socio-economic and COVID-19 specific factors, we find that, other things being equal, women had significantly lower levels of well-being than men;^11^ people in higher class positions and who have found themselves in similar or better financial positions reported higher scores of well-being as can be expected. Similarly, those who had suffered COVID-19 infections or received shielding letters also reported, as can be expected, lower levels of well-being. Other things being equal, keyworkers were feeling more satisfied with life. They may have felt proud of their selfless contributions they were making to the country.

Finally, we wish to explore two crucial questions: the relative importance between social capital versus demographic-socio-cultural factors in impacting on well-being; and the relative importance of social capital for well-being for the white group versus ethnic minority groups. To address this question would essentially mean conducting interactional analysis between types of social capital and ethnicity and compare the coefficients for the white group versus ethnic minority groups.

The data in Table 7 are conducted for this purpose. Firstly, we can see that the coefficients for the three types of social capital are very strong and mostly highly significant, which forms a sharp contrast to the coefficients of the demographic and socio-economic including COVID-19 specific variables, suggesting the usefulness of the social capital as we have constructed for this analysis. Among the latter, only the coefficients of financial situation were consistently predictive of people’s well-being. Secondly, we see that the coefficients vary somewhat among the different ethnic groups but without marked differences. We conducted pair-wise comparisons for all 36 pairs between each of the ethnic minority groups and the white group at each level for all three types of social capital, and only found three being significant: at the top two levels of social support, people of Pakistani/Bangladeshi origins have a significantly higher level of well-being than the white people (1.968 vs. 1.681; 0.98 vs. 0.787); and at the third level of social contact, people of “other” group had significantly higher level of well-being than the white group (0.125 vs. −0.160). These two features suggest that social capital played a crucial role on people’s well-being during the most devastating days of the COVID-19 period and that the roles were generally equally important for all ethnic groups.

**Table 7:** Regression coefficients on well-being by social capital, socio-demographic attributes, and ethnicity.

	White	Black	Indian	Pakistani-Bangladeshi	Other
**Social contact**					
Top	−0.370***	−0.285*	−0.322**	−0.394***	−0.231
2nd	−0.346***	−0.268*	−0.255*	−0.308***	−0.154
3rd	−0.160***	−0.107	−0.005	−0.085	**0.125**
**Social support**					
Top	1.681***	1.846***	1.785***	1.968***	1.719***
2nd	0.787***	0.905***	0.909***	0.980***	0.913***
3rd	0.609***	0.653***	0.775***	0.630***	0.539***
**Social trust**					
Top	0.703***	0.674***	0.755***	0.556***	0.836***
2nd	0.547***	0.499***	0.399***	0.374**	0.627***
3rd	0.414***	0.322***	0.339***	0.390***	0.326**
**Sex (male = ref)**					
Female	−0.138***	−0.109	−0.104	−0.127*	−0.155
Class (unskilled = ref)					
Higher salariat	0.056*	0.012	0.019	0.268	−0.011
Lower salariat	0.019	−0.127	−0.074	0.118	−0.071
Intermediate	0.042	−0.101	−0.022	0.162	0.108
Skilled manual	−0.089**	−0.332	0.065	0.148	−0.240
Workless	−0.111***	−0.196	−0.070	0.102	−0.240
**Financial change (worse = ref)**					
Same	0.116***	0.150	0.199**	0.212***	0.156
Better	0.086***	0.177	0.214**	0.349***	0.061
Having got COVID infection	−0.059***	−0.086	0.047	−0.013	0.143
Being a keyworker	0.075***	−0.014	0.212**	0.108	−0.068
Having received shielding letter	−0.194***	−0.034	0.033	−0.284*	−0.292
Constant	−0.953***	−0.951***	−1.254***	−1.338***	−1.173***
*R* ^2^	0.570	0.579	0.584	0.615	0.599
*N*	54,789	1,139	1,219	1,407	1,102

Pair-wise comparisons are conducted between the coefficients of the white group and those of the ethnic minority groups at each level of the three types of social capital, with significant differences (at the 0.05 level) shown in bold.

## Discussion

We have, in this paper, made a new effort at conceptualising and measuring social capital in the UK at the time of the coronavirus, linked it with people’s physical and mental well-being, and explored ethnic differences in terms of both social capital generation and well-being. We followed theoretical guidance in refining the conceptual and measurement work on social capital (Putnam, 2000; Lin, 2001; Li, 2015) but, given the special situation of the pandemic where civic participation in organisational activities was made infeasible by the lockdown and social distancing policies, we adjusted our measurement. We focused on domains of social capital unique to the pandemic situation: social contact people had with family or friends who did not live with them; social support they could obtain from family, friends and community members, socio-political trust they held in fellow citizens and in British Governments and political parties. We used 12 indicators for the three types of social capital, and a further six indicators for well-being. We used the latent response models (or item response theory, IRT, modelling) to “distil” the latent propensities of social capital and well-being which is most suitable for social surveys with categorical/ordinal indicator variables with missing data.

While conceptual vigour and methodological rigour are important, our main interest in this paper is in the substantive domain, namely, the ethnic and socio-economic differences in the different types of social capital, and the direct and indirect impacts of the different types of social capital on well-being. In each of these areas, we believe that our analysis has shed new light. Our main findings can be summarised as follows.

First, our latent response analysis shows that the indicator variables within each set were indeed associated with a single dominant underlying latent variable, and that the latent variables were rather weakly correlated with each other (Tables 1, 2). This indicates that we could measure domains of social capital under the pandemic situation as guided by theoretical considerations and that the domains were distinct, insusceptible to multilinearity problems. Our further analysis of the determinants of the three types of social capital reveals that apart from social contact where those from Black backgrounds were more likely to have higher scores of social contact than the white respondents, all ethnic minority groups had lower levels of social support, socio-political trust and well-being than the white respondents (Table 4), and the gross ethnic differences were again seen in their net effects when other socio-economic and COVID-19 specific factors were taken into consideration (Table 5). We also found, in Tables 4, 5, clear class gradients of social support, socio-political trust, and well-being.

To address the question of ethnic and social capital’s impacts on well-being during the pandemic, our findings in Table 6 show that when viewed independently, each type of social capital plays a positive role on people’s well-being, with social support and trust having more prominent impacts than social contact, and that ethnic minorities were disadvantaged in well-being. Yet when all three domains of social capital were simultaneously included in the model, the role of social contact on well-being became negative, which can be seen as a negative effect of making too much effort in helping others during the very hard times of the COVID-19 on one’s own physical and mental health, somewhat akin to the “dark side” of social capital as noted in previous literature (Portes, 1998; Putnam, 2000). As we have seen in Table 4, women have a score of 0.380 higher in social contact and 0.166 lower in well-being than do men, both highly significant at the 0.001 levels, showing the great sacrifices women were making.

Our analysis also shows persisting socio-economic differences. As shown above, people in the more advantaged positions were more likely than those in the lower positions to report improved financial situation, less vulnerability to COVID-19 infection, higher social support and trust, and higher levels of physical and psychological well-being. Class-lined resources are clearly manifested in social support and trust, and through them, in people’s well-being in the most challenging times of the COVID pandemic. Ethnic minority status and socio-economic disadvantage often go hand in hand, particularly for people from Pakistani/Bangladeshi and Black origins who, as found in numerous other studies, tend to have poorer employment opportunities, lower class positions, worse economic conditions, greater vulnerability to COVID infection and lower levels of well-being. In sum, they have multiple disadvantages.

Our analysis in Table 7 shows the marked effects of social capital on well-being relative to demographic and socio-economic including the pandemic-specific variables and that there were generally no ethnic specific differences in the roles played by social capital. Thus the source of ethnic disparity lies in the socio-economic disadvantages which manifest themselves in the different profiles of social capital with salient impacts on well-being but for people with similar levels of social capital and socio-economic conditions, there were no pronounced ethnic-specific effects. The root cause of disadvantage thus lies in socio-economic inequality rather than ethnicity but ethnicity is a notable bearer of such inequality.

Our analysis has limitations. There are many specific questions that we would have wished to but could not address either because the information is not available in the survey, or because they were asked only in one wave or another but not across all three waves or were only asked to specific cohorts. For instance, there may be complicated mechanisms for the social capital generation and the multiple disadvantages by different ethnic minority groups which affect their well-being levels, which we could not fully explore due to data limitation.

There are two main questions awaiting future research. Firstly, how resilient is social capital beyond the pandemic? Has community life survived and returned to the pre-COVID level? Is the post-COVID social capital as class-lined as before? What is the relative role of formal and informal social connections for people’s well-being? We are now in a turbulent world with the wars going on in Ukraine and Gaza, and with the living costs soaring high. In such a situation, how strong is the social fabric that binds our community together such as our charitable behaviour in giving time, effort and money to the weak, poor and needy? The current data cannot provide answers to such questions as the needed information was not collected. The second question pertains to ethnicity. Some of our cohort members were rather old and when the earlier cohort surveys were designed such as that of the 1946 NSHD, the 1958 NCDS, and the 1970 BCS, there was only a small proportion of ethnic minorities in the population and there were no standard and detailed ethnic classifications available at that time. The ethnic framework used in this paper is rather crude, with only five categories. We need data with more elaborate ethnic coding to do a more systemic analysis on ethnic differences to reflect the ethnic composition as currently found in the UK. We await new data for such analysis, to push forward the frontiers of knowledge.

## Data availability statement

Publicly available datasets were analyzed in this study. This data can be found here: https://www.data-archive.ac.uk, SN = 8658.

## Author contributions

YL conducted the analysis and wrote the paper. LD prepared the files and joined the discussion. YL and LD read the manuscript and approved it for publication. All authors contributed to the article and approved the submitted version.

## Funding

This work received no specific funding but benefited from the first author’s previous funded projects as PI or Co-PI such as ‘Socio-Economic Position of the Minority Ethnic Groups in Britain’ (RES-163-25-0003), ‘Social Mobility and Social Capital in China and Britain’ (ES/I035168/1), ‘Understanding the Dynamics of Ethnic Identity and Inequality in the UK’ (ES/J020036/1), and ‘Social Complexity of Immigration and Diversity’ (EP/H02171X/1).

## Footnotes

^1^We are grateful to a Reviewer for the insight in this regard.

^2^There are various studies on social capital during the COVID-19 period or more generally in times of public crises, such as Bartscher et al. (2021), Jean-Baptiste et al. (2020). But little is known about how ethnic groups use different types of social capital during the most challenging times of COVID-19 in the UK.

^3^We are grateful to one of the Reviewers who suggested that we provide more information on the surveys. As the technical details are rather complicated and are very well documented in Brown et al. (2020), we would refer interested readers to this important document for a good understanding of the survey methodology. The questionnaires of the surveys are available at the data-archive.ac.uk (Serial Number = 8,658).

^4^The White group includes White British, White Irish, White Other; the Black group includes Black Caribbean, Black African and Black Other; and ‘Other’ group includes Chinese, Mixed and various other small ethnic groups.

^5^For substantive analysis from Table 2 onward in this paper, we use weighted analysis. The source file contains a weight variable called cw2_combwt which contains a value of −1 for MCS parents (N = 13,777). As negative values are not allowed for weighted analysis, we consulted Professor Richard Silverwood at UCL’s Centre for Longitudinal Studies, who advised us to use a value of 1 for the MCS parents. We wish to express our gratitude for his advice here.

^6^Further analysis shows that ethnic minority groups have lower scores than do white respondents in both generalized and political trust. With regard to generalized trust, the mean scores (from 0–10 with 10 indicating complete trust) for white, Black, Indian, Pakistani/Bangladeshi and Other groups are 6.3, 5.6, 6.1, 5.9 and 6.0, and scores for trust in British government and political parties range from 4.1, 3.0, 3.9, 3.3 to 3.5, respectively. These echo findings of lower generalized trust by ethnic minority groups than by white people in Britain from 2007 to 2017 (Li et al., 2018: 7). The persisting low levels of social trust by ethnic minorities may reflect the hardships they experienced in the labour market in terms of unemployment, hyper-cyclical unemployment and discrimination (Cheung and Heath, 2007; Heath and Di Stasio, 2019; Li, 2021).

^7^Further analysis, using Wave 1 data for example, shows that men tend to have more formal work hours than women, at 17.9 and 11.7 h per week including overtime, but women tend to spend more hours than men on home schooling children, other interactive activities with children, caring for someone other than a child and housework, at 38.3 and 24.5 h per week, respectively. Overall, the more formal and informal work hours might have put a strain on women’s well-being. We wish to thank one of the reviewers for this insight.

^8^It is noted here that people in higher class positions were much more likely than those from routine working-class positions to find their family financial position had turned better since the COVID-19 outbreak: 29.9% of the respondents in higher salariat as against 15.6% for the routine workers reported improved financial position.

^9^Further analysis shows that keyworkers were more likely to catch Coronavirus (14.6% as compared with 10.6% for non-keyworkers). They were less likely to suffer financial deterioration due to the pandemic (17.9% versus 25.1% for the non-keyworkers reporting ‘worse off’).

^10^Further analysis shows that people of Black heritages are 11.4% behind the white group in generalised trust and 24.6% behind the white respondents in political trust. In other words, the Black community are over twice as distrustful of the Government and political parties as the general population.

^11^Further analysis shows significant class*gender but little cohort*gender differences: women in each class tend to have significantly lower scores of well-being than their male peers but only women in the BCS have a slightly higher score of well-being than their male peers (b = 0.044, *p* = 0.046). We wish to thank one of the Reviewers for the suggestion.
